# Serous cystadenoma of the pancreatic head associated with pancreatic duct atresia and acinar cell loss: a case report

**DOI:** 10.1186/s12893-026-03772-9

**Published:** 2026-05-01

**Authors:** Ting-Yu Yang, Ming-Dong Chen, Ai-Ping Huang, Qian-Jun Yu, Zhi Zhong, Chuan Zhao, Chun-Lin Deng, Long Cheng

**Affiliations:** 1 North Sichuan Medical College, Nanchong, China; 2 Department of General Surgery, The General Hospital of Western Theater Command, Chengdu, Sichuan P.R. China

**Keywords:** Serous cystadenoma, Pancreatic duct atresia, Acinar cell loss, Pancreaticoduodenectomy

## Abstract

**Background:**

Serous cystadenoma (SCA) of the pancreas is typically a benign neoplasm, and variants are exceptionally rare. While tumors in the pancreatic head often cause upstream ductal dilatation and parenchymal atrophy due to obstruction, the concurrent presentation of SCA with congenital distal pancreatic duct atresia and complete acinar cell loss is unique. We report this case to highlight a potential congenital pathogenesis linking ductal developmental defects to the formation of these lesions.

**Case presentation:**

A 56-year-old woman presented with persistent right upper abdominal distension and discomfort lasting over one year. She had a 20-year history of an abdominal mass and had undergone two previous unsuccessful surgical interventions, including a palliative bypass and an aborted resection. Preoperative imaging revealed a large cystic-solid mass in the pancreatic head. Intraoperative exploration identified a 12-cm lesion replacing the pancreatic head and neck, accompanied by complete fatty replacement of the pancreatic body and tail. The patient underwent en bloc pancreaticoduodenectomy with partial portal vein resection and synthetic vascular graft reconstruction. Histopathological examination confirmed the diagnosis of SCA. Notably, the distal pancreatic tissue exhibited a complete absence of acini and visible ductal structures, while islet cells were preserved. Postoperative recovery was uneventful, and at the 12-month follow-up, the patient remained disease-free with preserved endocrine function despite the loss of exocrine function.

**Conclusions:**

This case illustrates the rare coexistence of a SCA with congenital distal pancreatic duct atresia and acinar cell depletion. The preservation of islet function amidst complete exocrine loss supports a developmental anomaly rather than obstruction-induced atrophy. This association suggests that congenital ductal defects may predispose patients to SCA formation. Surgical resection remains the optimal strategy for the diagnosis and management of atypical cystic pancreatic lesions with uncertain biological behavior.

## Background

Serous cystadenoma (SCA) is a rare, typically benign cystic neoplasm comprising 1–2% of all pancreatic tumors, occurring mainly in women and often discovered incidentally [1–3]. Histologically, SCAs are composed of glycogen-rich epithelial cells derived from centroacinar cells [4]. Pancreatic head tumors often cause obstruction and upstream ductal dilatation with parenchymal atrophy [5]. However, the coexistence of pancreatic head tumor with distal ductal atresia and complete acinar loss is extremely uncommon. Here, we present a unique case of SCA of the pancreatic head with distal pancreatic duct atresia and complete fatty replacement of the body and tail, and we discuss its potential congenital pathogenesis.

## Case presentation

**Fig. 1:**
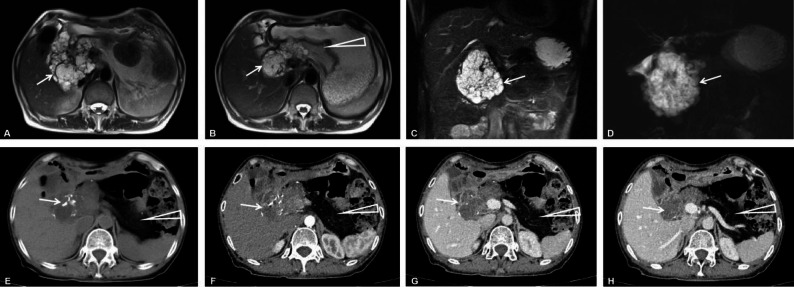
Imaging features. (**A**, **B**) Axial magnetic resonance images demonstrate a cystic-solid mass located at the hepatic hilum and pancreatic head, exhibiting a multicystic pattern with visible septations (↑). (**C**) Coronal magnetic resonance image showing a cystic-solid mass at the hepatic hilum and pancreatic head, also presenting a multicystic pattern with septations (↑). (**D**) MR cholangiopancreatography shows no visualization of the main pancreatic duct. (**A**) Plain CT scan reveals a mixed-density mass with internal calcifications. (**B**) Arterial phase CT shows mild enhancement of the lesion. (**C**, **D**) Venous phase delayed enhancement images reveal a multilocular cystic mass in the pancreatic head, composed of numerous small locules with lobulated contours, and central enhancing calcifications (↑). The pancreatic body and tail region is completely replaced by fatty tissue without identifiable pancreatic duct (△)

**Fig. 2:**
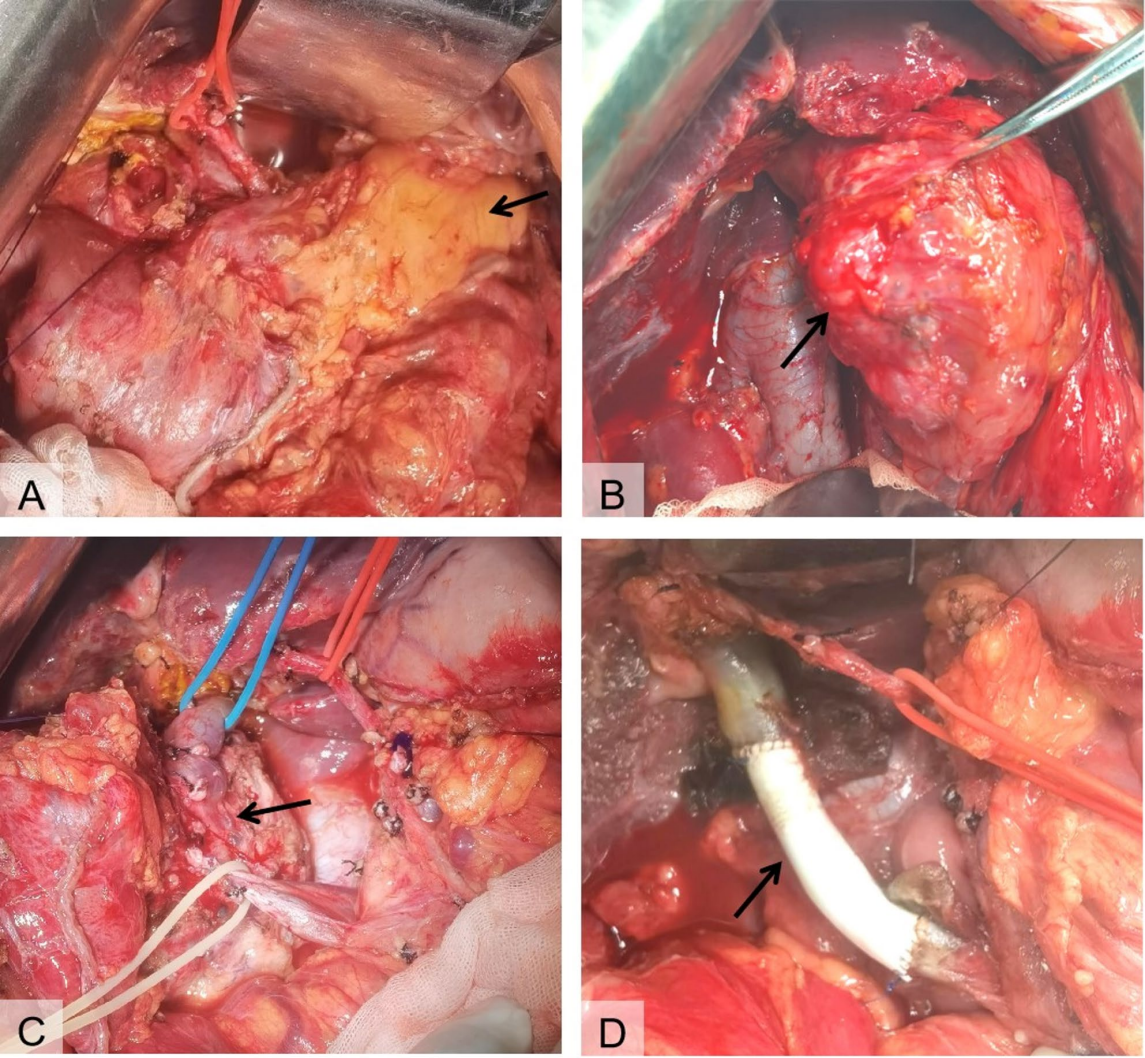
Intraoperative findings. **A** Fatty replacement of the pancreatic body and tail (↑). **B** Large mass occupying the pancreatic head (↑). **C** Tumor adhesion to the portal vein and superior mesenteric vein (↑). **D** En bloc resection of the tumor and involved vessels, followed by artificial vascular graft reconstruction (↑)

**Fig. 3:**
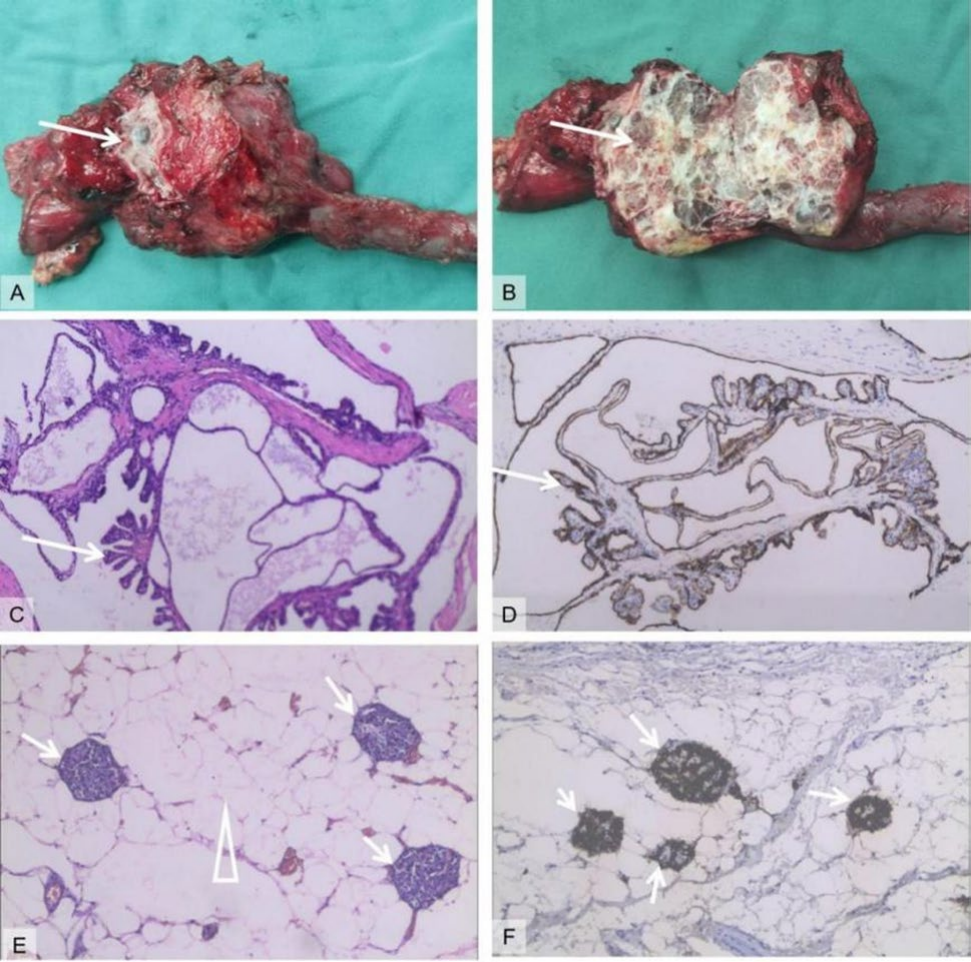
Pathological examination. **A** Gross specimen demonstrating tumor adhesion to the portal vein and superior mesenteric vein (↑). **B** Cut surface of the tumor showing a multicystic architecture with focal calcification (↑). **C** Hematoxylin-eosin (H&E) staining revealing cystic structures lined by columnar epithelium. **D** Immunohistochemistry showing CK19 positivity in the lining epithelial cells. **E** H&E staining of adjacent pancreatic tissue demonstrating fatty replacement (△) with scattered islet cell clusters but no acinar structures. **F** INSULIN immunostaining exhibiting strong labeling of islet cell clusters (↑)

**Fig. 4:**
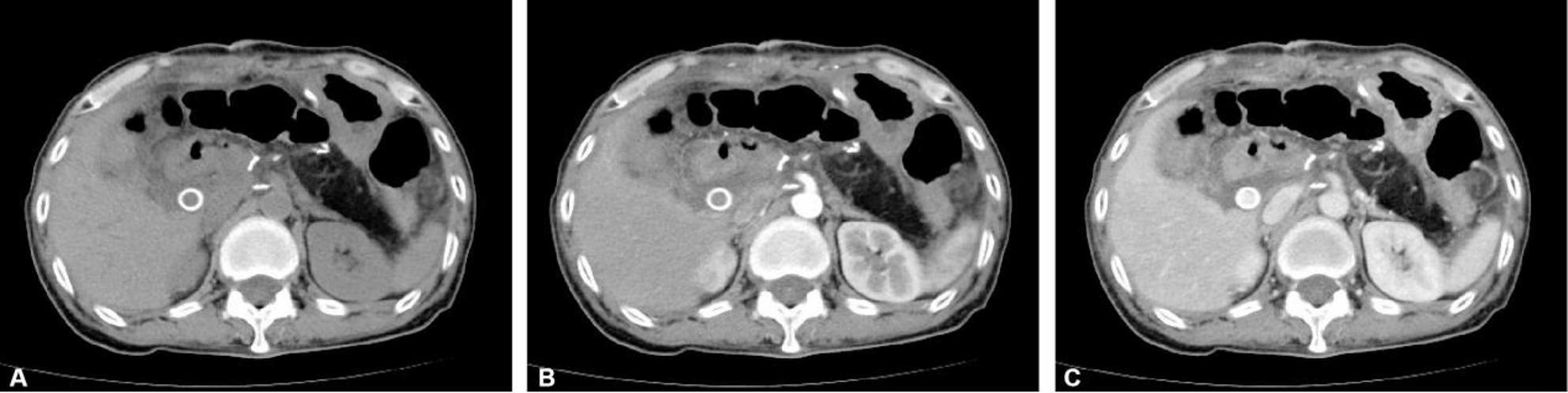
Follow-up CT scan at 1 year postoperatively showing no tumor recurrence, no local fluid collection, patent vascular graft, and preserved hepatic perfusion

A 56-year-old woman presented with right upper abdominal distension and discomfort lasting over a year. The patient had a 20-year history of an abdominal mass and a complex surgical background involving two previous unsuccessful interventions, namely a palliative bypass and an aborted resection. Twenty years prior, she underwent a palliative biliary and gastric bypass consisting of a cholecystectomy, choledochojejunostomy, and gastrojejunostomy for a suspected pancreatic head tumor; however, tumor resection was not performed at that time, and no biopsy was taken. Five years prior to the current admission, an attempted resection at another institution was aborted intraoperatively due to severe vascular adhesions. Thus, no prior pathological diagnosis was available until the current surgery. Physical examination revealed a firm epigastric mass approximately 10 cm in diameter with limited mobility. Laboratory results, including tumor markers, were normal; fasting glucose and HbA1c were within reference ranges. Contrast-enhanced CT showed a cystic-solid mass in the pancreatic head with enhancing septa and calcifications, and MRI confirmed an irregular multilocular cystic lesion without ductal communication (Fig. 1). Endoscopic ultrasound demonstrated a 10-cm exophytic cystic-solid lesion from the pancreatic neck. Exploratory laparotomy revealed extensive adhesions. The pancreatic head and neck were completely replaced by tumor, which measured over 10 cm in diameter, while the body and tail were entirely fatty. The pancreas was transected left of the lesion; no normal parenchyma or duct was seen. Given the absence of pancreatic juice outflow, the stump was closed directly. Due to dense inflammatory adhesions between the tumor and the vascular wall, an en bloc resection of the tumor with segments of the portal and superior mesenteric veins was performed, followed by synthetic graft reconstruction and regional lymphadenectomy (Fig. 2). Operative time was 8 h; blood loss was 600 mL. Grossly, the 10 × 8 × 8 cm tumor was multicystic with focal calcifications. Microscopically, the tumor demonstrated cystic structures lined by flat to cuboidal epithelial cells with clear cytoplasm containing glycogen, consistent with SCA. The adjacent pancreatic tissue showed complete fatty replacement with scattered islet cell clusters but no identifiable acinar structures or ducts. Immunohistochemistry confirmed CK19 positivity in the lining epithelial cells and insulin positivity in the preserved islet clusters (Fig. 3). The patient’s postoperative course was uneventful. At the 12-month follow-up, CT scan showed no tumor recurrence, a patent vascular graft, and preserved hepatic perfusion (Fig. 4). Postoperative endocrine assessment demonstrated preserved beta-cell function with appropriate glucose-stimulated insulin and C-peptide responses (Table 1). The patient required pancreatic enzyme supplementation for exocrine insufficiency but maintained normal glucose tolerance without medication.

**Table 1:** Postoperative assessment of pancreatic endocrine function

Time Point	Serum Glucose (mmol/L)	Serum C-Peptide (nmol/L)	Serum Insulin (µU/mL)
Fasting	5.39	0.44	2.68
30 min	7.5	0.73	6.75
60 min	10.19	1.09	11.27
120 min	11.56	1.88	28.27
180 min	9.91	2.38	19.94

HbA1c: Glycosylated hemoglobin. The patient’s HbA1c level was 6.3% (Reference range: 4.0%–6.5%). Autoantibodies: The diabetes autoantibody panel, including Glutamic Acid Decarboxylase Antibody (GADA), Insulin Autoantibody (IAA), Tyrosine Phosphatase Antibody (IA-2 A), and Zinc Transporter 8 Antibody (ZnT8A), was negative

## Discussion

Serous cystic neoplasm (SCN) of the pancreas, also known as serous cystadenoma, represents a distinct entity among pancreatic cystic lesions. Our case presents a unique constellation of findings: a large SCA of the pancreatic head associated with distal pancreatic duct atresia and complete acinar cell loss, while islet function remained preserved. This unusual combination raises important questions about the potential relationship between congenital pancreatic ductal anomalies and the development of serous cystic tumors.

The differential diagnosis of pancreatic fatty replacement includes several entities. Lipomatous pseudohypertrophy of the pancreas, as described by Yasuda et al. [6], is characterized by fatty infiltration with preservation of pancreatic architecture. In our case, the complete absence of acinar tissue and ductal structures, with selective preservation of islet cells, differs from typical lipomatous pseudohypertrophy. The pattern observed is more consistent with congenital ductal atresia leading to agenesis of exocrine elements while endocrine components, derived from a different developmental pathway, remain intact.

Regarding the embryological considerations raised by reviewers, the pancreas develops from two separate anlagen: the dorsal bud, which gives rise to the body, tail, and part of the head, and the ventral bud, which forms the remainder of the head and uncinate process. During normal development, differential growth and fusion occur between these two primordia. It is possible for developmental anomalies to affect primarily the dorsal pancreas while sparing the ventral portion [7]. In the context of our case, even if the distal pancreas (derived from the dorsal bud) exhibited developmental abnormalities including ductal atresia and acinar agenesis, the pancreatic head region (derived predominantly from the ventral bud) could still contain normal ductal epithelium and centroacinar cells. These ventrally derived cells could serve as the origin for SCA development. This embryological explanation addresses the apparent paradox of SCA arising from centroacinar cells despite distal acinar absence.

We acknowledge that distinguishing between congenital and acquired etiologies remains challenging. The massive tumor size and associated chronic inflammation made evaluation of anatomical structures at the tumor boundary difficult. Both mechanisms could potentially explain the observed findings: congenital ductal atresia leading to lipomatous atrophy, or secondary fatty replacement from long-standing tumor obstruction. The preserved islet function with normal glucose metabolism and appropriate insulin/C-peptide responses at 12-month follow-up provides some support for a congenital mechanism, as purely secondary atrophy might be expected to affect both exocrine and endocrine components more uniformly. However, we recognize that this evidence is not definitive, and further investigation with additional similar cases would be valuable to clarify this relationship.

Complete fatty replacement of the pancreas is an uncommon finding. Schulz et al. [8] reported a giant serous microcystic adenoma with 20 years of follow-up, though without the associated ductal atresia seen in our case. The distinction between congenital and acquired causes of pancreatic lipomatosis has important implications for understanding tumor pathogenesis. If congenital ductal atresia predisposes to SCA formation, this would suggest a novel developmental pathway linking ductal anomalies to serous cystic tumor development.

Surgical management of large pancreatic head tumors with vascular involvement remains challenging. In our case, en bloc resection with portal vein reconstruction was necessary due to dense inflammatory adhesions. When vascular invasion cannot be excluded preoperatively, preparation for vascular reconstruction is essential. The decision to proceed with extended resection is justified by the uncertainty regarding the tumor’s biological behavior and the potential for curative treatment. Even when the final pathology confirms a benign lesion, complete resection provides definitive diagnosis and prevents complications from continued tumor growth.

The preservation of endocrine function despite complete exocrine loss is noteworthy. This finding has been reported in cases of pancreatic lipomatosis and supports the hypothesis of differential developmental origins for exocrine and endocrine components [7]. The clinical implications include the need for pancreatic enzyme replacement while reassuring patients regarding their long-term diabetic risk. In our patient, normal glucose tolerance with preserved insulin secretory capacity was maintained at 12-month follow-up.

Several limitations of our report should be acknowledged. First, the retrospective nature and single-case design preclude definitive conclusions about causality between ductal atresia and SCA development. Second, the extensive surgical adhesions and chronic inflammation limited our ability to fully evaluate the anatomical relationship between the tumor and surrounding pancreatic parenchyma, including the status of the minor papilla and accessory pancreatic duct. Third, distinguishing between congenital and secondary fatty replacement based on histopathology alone is challenging. Future studies with more cases would be valuable to explore this proposed association.

## Conclusions

This exceptional case of SCA with distal pancreatic duct atresia and acinar loss illustrates a possible congenital pathogenesis underlying serous cystic tumors. The embryological development of the pancreas, with separate dorsal and ventral anlagen, may explain how SCA can arise from centroacinar cells in the pancreatic head despite distal developmental abnormalities. Awareness of this association aids accurate diagnosis and appropriate surgical management.

## Data Availability

The datasets generated and analyzed during the current study are available from the corresponding author on reasonable request.

## Abbreviations

SCA: Serous cystadenoma
CT: Computed tomography
MRI: Magnetic resonance imaging
H&E: Hematoxylin-eosin
BMI: Body mass index
